# Incidence and clinical features of patients with peritoneal dialysis peritonitis complicated by bacteremia

**DOI:** 10.1097/MD.0000000000013567

**Published:** 2018-12-10

**Authors:** Chang-Chih Tsai, Chien-Chin Hsu, Kuo-Tai Chen

**Affiliations:** a Emergency Department, Chi Mei Medical Center; b Department of Biotechnology, Southern Tainan University of Technology; c Department of Emergency Medicine, School of Medicine, College of Medicine, Taipei Medical University, Taipei, Taiwan.

**Keywords:** bacteremia, clinical features, outcome, peritoneal dialysis, peritonitis

## Abstract

The standard treatment of peritoneal dialysis peritonitis (PD peritonitis) is intraperitoneal antibiotic therapy. In patients with PD peritonitis complicated by bacteremia, intraperitoneal antibiotics combined with elective removal of the infected intraperitoneal catheter may be inadequate.

We collected data of all patients with PD peritonitis admitted to Chi-Mei Medical Center during a 4-year period. We reviewed the medical records of the study cohort and collected their in-hospital details. Patients with positive blood culture results were assigned to the bacteremia group, whereas those with negative blood culture results were assigned to the peritonitis-only group.

We discovered that 11.0% of patients with PD peritonitis had bacteremia complications, and immunocompromised comorbidities were more common in the bacteremia group than in the peritonitis-only group (66.7% vs 37.2%, *P* = .022). Additionally, the bacteremia group exhibited higher temperatures, greater respiratory rates, and lower serum sodium levels than the peritonitis-only group (temperature, 37.7 vs 37.2 °C, *P* = .014; respiratory rate, 19.1 vs 17.9 rate/min, *P* = .008; serum sodium level, 130.3 vs 132.7 mEq/L, *P* = .031). No mortality was found in patients with PD peritonitis complicated by bacteremia after intravenous and intraperitoneal antibiotic therapy.

More than 1 in 10 patients with PD peritonitis was complicated by bacteremia, which resulted in extensive systemic derangements. Patients with immunocompromised comorbidities carried a higher risk of developing bacteremia, resulting in prolonged hospital stays. Combination of intraperitoneal and intravenous antibiotics therapies achieved fair prognoses in patients with PD peritonitis complicated by bacteremia.

## 1 Introduction

Peritoneal dialysis (PD) is a secondary common form of renal replacement therapy.^[1]^ The total population of PD recipients worldwide was estimated to be 27,200 in 2013, and the prevalence of PD is higher in developing countries than in developed countries.^[1]^ In patients treated with PD, infection is the primary cause of death; PD-related peritonitis (PD peritonitis) is the most common infectious disease, resulting in technique failure, transfer to hemodialysis, hospitalization, and death.^[1–3]^ PD peritonitis is regarded as a focal infection, and the standard treatment of PD peritonitis is intraperitoneal antibiotic therapy, which has demonstrated an advantage over intravenous antibiotic therapy.^[2,3]^ However, intraperitoneal antibiotic therapy exerts only a local effect on peritonitis and may not be suitable for the treatment of systemic infection. For patients with PD peritonitis complicated by bacteremia, traditional management (intraperitoneal antibiotics combined with elective removal of the infected intraperitoneal catheter) may be inadequate. Additionally, the incidence, presentation, treatment, and outcome of PD peritonitis complicated by bacteremia are still unknown. Accordingly, we conducted this retrospective study to determine the differences between PD peritonitis cases with and without bacteremia complication.

## 2 Methods

This study was reviewed and approved by the Institutional Review Board for Human Research at Chi-Mei Medical Center. We searched the emergency department (ED) discharge charts of Chi-Mei Medical Center for diagnoses of PD peritonitis (as defined by the Ninth Revision of the International Classification of Diseases code 996.6) from January 1, 2012, to December 31, 2015. In the study hospital, patients with suspected cases of PD peritonitis found in outpatient clinics were usually referred to the ED for rapid diagnosis and initiation of treatment. When peritonitis is suspected, dialysis effluent should be drained and sent for cell count with differential and culture. The nurses inoculated 5 to 10 mL effluent taken from suspected patients in 2 blood-culture bottles. If the ED doctors ordered blood culture investigations, the ED nurses also draw 10 mL blood twice from 2 different veins on each patients for blood culture investigations. Intraperitoneal antibiotics treatment was initiated after proper sampling. Intravenous antibiotics treatment was given on a patient only when positive result of his/her blood culture is presented. In this study, collected cases satisfying either of the following 2 criteria were further reviewed for diagnoses of PD peritonitis: positive ascites culture; or dialysate white cell count higher than 100 cells/μL, with at least 50% polymorphonuclear cells.^[3,4]^ Patients with diagnoses that met one of these 2 criteria were included in the study cohort. We reviewed every patient's hospital course and excluded patients with intra-abdominal infections other than PD peritonitis because secondary peritonitis has a higher mortality rate.^[5]^

We reviewed the medical records of the study cohort from the ED during hospitalization to collect the following data: general characteristics (age; gender; immunocompromised comorbidities including diabetes mellitus, liver cirrhosis, autoimmune diseases, and cancers; and long-term steroid requirements), vital signs in the ED (temperature, heart rate, respiratory rate, and systolic arterial pressure), laboratory tests (white blood cell counts of effluent and blood, serum glucose, sodium, potassium, C-reactive protein, alanine aminotransferase, and results of blood and dialysate cultures), and hospital courses and outcomes (length of hospital days; requirement of intensive care and removal of the peritoneal catheter; rate of recurrent, relapsing, repeat, or refractory peritonitis; shift to hemodialysis therapy; and mortality). The definitions of recurrent, relapsing, repeat and refractory peritonitis are listed. Recurrent describes an episode within 4 weeks of the completion of therapy for a previous episode, but with a different organism. Relapsing describes an episode within 4 weeks of the completion of therapy for a previous episode with the same organism or 1 sterile episode. Repeat describes an episode more than 4 weeks after the completion of therapy for a previous episode with the same organism. Refractory describes failure to respond to appropriate antibiotics within 5 days.^[4]^ The first and second authors completed all the chart review. Disagreement between authors were resolved by consensus, and if necessary, consultation with the third author.

We further evaluated those patients who had undergone blood culture investigations in the ED. Patients with positive blood culture results were placed in the bacteremia group, and those with negative blood culture results were placed in the peritonitis-only group. The collected variables were compared between the bacteremia and peritonitis-only groups.

### 2.1 Statistical analysis

Statistical analyses were performed using SPSS 15 (SPSS, Inc., Chicago, IL). We employed the chi-squared test to evaluate differences in the categorical variables and Student *t* test to evaluate differences in the continuous variables between the bacteremia and peritonitis-only groups. Continuous data results are presented as the mean ± standard deviation. In all cases, a *P* value <.05 was employed as the threshold for statistical significance.

## 3 Results

### 3.1 Characteristics, vital signs in the ED, and laboratory tests

From the 4-year study period, 180 cases were reviewed. The age range of the relevant patients was 20 to 91 years (mean, 54 years). The mean length of hospital stay of all patients with PD peritonitis was 9.3 ± 8.9 days, and intensive care was required in 12 patients (6.7%). Surgery to remove the dialysis tube was performed in 22.2% of patients, and 20.0% of patients were ultimately shifted to hemodialysis. The rates of recurrent, relapsing, repeat and refractory PD peritonitis within the study cohort were 4.4%, 7.2%, 11.1%, and 26.1%, respectively. A total of 8 patients (4.4%) in the cohort died during hospitalization.

**Figure 1:**
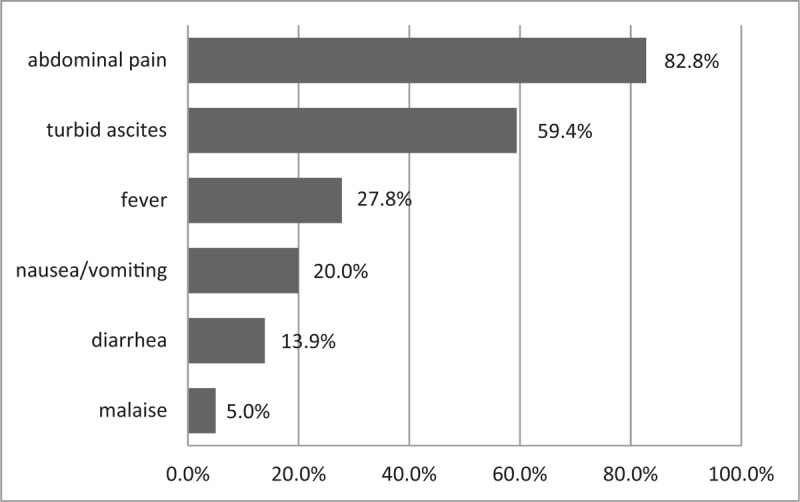
Common presenting symptoms at the emergency department.

Abdominal pain (82.8%) was the most common presenting symptom. Other clinical manifestations included turbid ascites (59.4%), fever (27.8%), nausea or vomiting (20.0%), diarrhea (13.9%), and malaise (5.0%) (Fig. 1). On presentation at the ED, 41.1% and 21.1% of patients exhibited tachycardia (heart rate >100 bmp) and fever (temperature ≥ 38°C), respectively. Tachypnea (respiratory rate >20 breaths/min; 5.0%) and hypotension (systolic arterial pressure <90 mmHg; 2.8%) were among the less common abnormal vital signs. The initial white cell count of the effluent was less than 100 cells/μL in 10 cases (5.7%). The majority of patients with PD peritonitis had elevated inflammatory markers (63.5% of cases with a white cell count >10,000 cells/μL and 80.2% with C-reactive protein >5 mg/L). Furthermore, patients with PD peritonitis exhibited physiological derangements including hypernatremia or hyponatremia (68.6%; serum sodium >148 or <135 mEq/L), hyperkalemia or hypokalemia (61.4%; serum potassium >5 or <3.5 mEq/L), abnormal random serum glucose (14.9%; >250 g/L or <70 g/L), and abnormal serum alanine aminotransferase (14.4%; >40 U/L).

### 3.2 Differences between bacteremia and peritonitis-only groups

A total of 167 patients had undergone blood culture investigations in the ED, and 22 tested positive for bacteremia. We excluded 4 of these 22 patients because the pathogens isolated from blood cultures were different from pathogens isolated from the effluent. In 1 patient, the bacteremia was assumed to be derived from concomitant foot necrotizing fasciitis, because the pathogens isolated from blood cultures were similar to the pathogens isolated from the wound culture. The positive blood culture results in the other 3 patients were interpreted as contamination.

**Figure 2:**
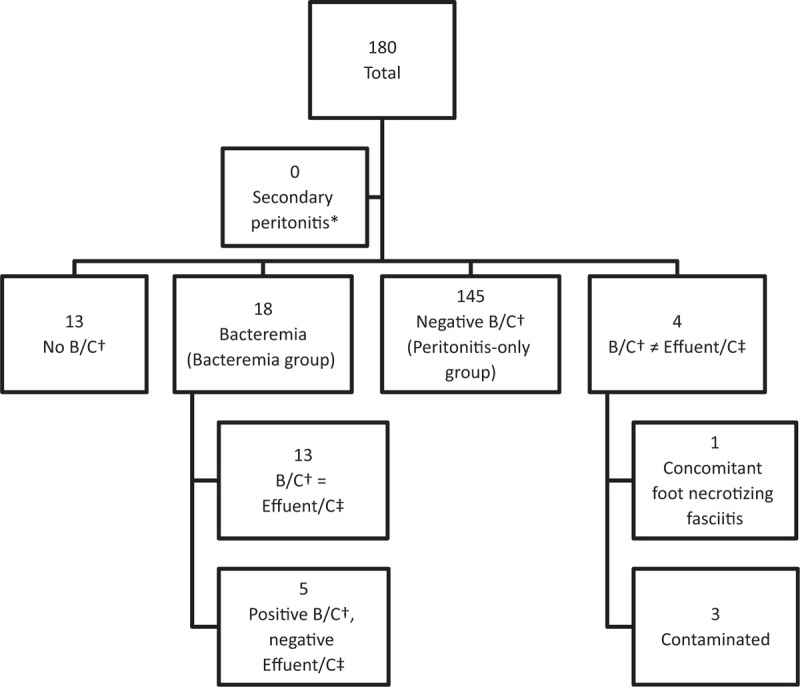
Categories according to the results of blood culture and effluent culture. ∗ Patients with intra-abdominal infections other than peritoneal dialysis peritonitis. † Blood culture, ‡ Effluent culture.

Among the remaining 18 patients, 13 presented identical blood culture results and effluent cultures. Another 5 showed positive blood culture results with negative effluent culture results, and we did not discover an infection source other than PD peritonitis. Thus, these 18 patients were placed in the bacteremia group, and patients with negative blood culture results were placed in the peritonitis-only group (n = 145). All patients in both groups underwent intraperitoneal antibiotic therapy, and the choice of antibiotics was based on the results of the effluent culture or, when the effluent culture results were negative, empirical selection by the nephrologist. Every patient in the bacteremia group was administered additional intravenous antibiotics. (Fig. 2)

Comparing the bacteremia group with the peritonitis-only group, we identified no differences in age or gender. The incidence of immunocompromised comorbidities was higher in the bacteremia group (66.7% vs 37.2%, *P* = .022). The bacteremia group exhibited higher temperatures, greater respiratory rates, and lower serum sodium levels in the ED than the peritonitis-only group (temperature, 37.7 vs 37.2°C, *P* = .014; respiratory rate, 19.1 vs 17.9 rate/min, *P* = .008; serum sodium level, 130.3 vs 132.7 mEq/L, *P* = .031). After intravenous and intraperitoneal antibiotic therapy, the overall prognoses of patients with PD peritonitis complicated by bacteremia were fair and the mortality rate was low. Subsequently, compared with the peritonitis-only group, the bacteremia group exhibited more intensive care requirements, more dialysis catheter removals, and more hemodialysis therapy. Furthermore, the bacteremia group had longer hospital stays than the peritonitis-only group (hospital stay, 13.5 vs 8.6 days, *P* = .011) (Table 1).

**Table 1:**
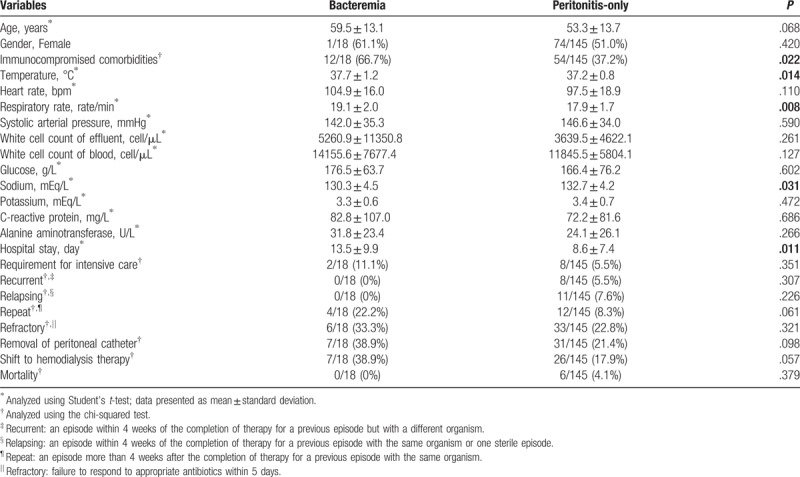
Characteristics, vital signs in the emergency department (ED), laboratory tests, and hospital courses; outcomes of the bacteremia and peritonitis-only groups.

### 3.3 Bacteriologic analysis

Gram-positive cocci constituted 57.7% of all infectious organisms in the effluent culture, and *Streptococcus* spp. were the most common isolated gram-positive cocci, followed by *Staphylococcus aureus*, *Coagulase-negative Staphylococci*, and *Enterococcus* spp. Gram-negative bacilli constituted 38.0% of infectious organisms, and *Escherichia coli* was the most common isolated gram-negative bacillus. Other infections found included 2 *Actinobacter* spp., 2 *Candida* spp., and 2 *Mycobacterium* spp. infections (Table 2). Polymicrobial infections were identified in 7 patients (3.9%), and negative effluent cultures were discovered in 39 patients (21.7%).

**Table 2:**
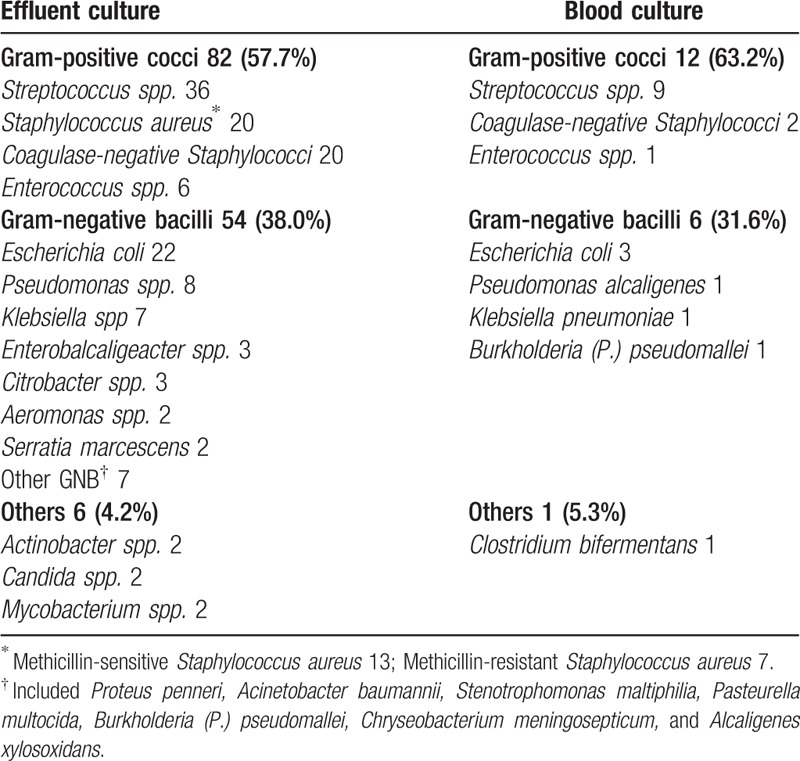
Isolated bacteria from effluent and blood cultures.

The incidence of bacteremia was 11.0% (18/163), and the blood cultures in the 18 patients with bacteremia yielded 19 infectious pathogens. Gram-positive cocci constituted 63.2% of all infectious organisms and included 9 *Streptococcus* spp., 2 *Coagulase-negative Staphylococci*, and 1 *Enterococcus*. Gram-negative bacilli constituted 31.6% of infectious organisms and included 3 *E. coli*, 1 *Pseudomonas alcaligenes*, 1 *Klebsiella pneumoniae*, and 1 *Burkholderia (P.) pseudomallei*. One anaerobic infection (*Clostridium bifermentans*) constituted 5.3% of infectious organisms (Table 2).

## 4 Discussion

We discovered that 11.0% of patients with PD peritonitis had bacteremia complications, and bacteremia resulted in extensive systemic derangements such as fever, tachypnea, and abnormalities in serum sodium levels. The patients in the bacteremia group presented with higher disease severity (longer hospital stay). Lee et al reported successful treatment of a patient with PD peritonitis complicated by bacteremia using combined intravenous and intraperitoneal antibiotics.^[6]^ We followed this regimen for our patients with PD peritonitis complicated by bacteremia and obtained favorable outcomes. Traditionally, PD peritonitis is regarded as a focal infection, and blood culture is not regularly taken from patients with PD peritonitis. Most patients with PD peritonitis undergo outpatient treatment, which is unsuitable for patients with bacteremia complications.^[7–9]^ A retrospective study conducted in Taiwan revealed that PD peritonitis is the most common cause of bacteremia in patients undergoing PD.^[9]^ Accordingly, physicians should develop a more effective method of identifying bacteremia complications in patients with PD peritonitis. Given that more than 1 in 10 patients with PD peritonitis has bacteremia complications, we suggest that all patients with PD peritonitis consider blood culture investigation, especially those with immunocompromised comorbidities.

The patients with PD peritonitis in this study usually presented to the ED with gastrointestinal symptoms and turbid ascites. On arrival, tachycardia and fever were common abnormal vital signs. Physicians should be aware of these symptoms and always consider the possibility of PD peritonitis in patients with end-stage renal disease who are undergoing PD therapy. Some of these patients cannot express their symptoms properly because of preexisting comorbidities (cerebral vascular accident, dementia, aphasia) or depressed consciousness due to sepsis.^[10]^ Some authors have even suggested that effluent analysis and culture be drawn from all patients undergoing PD that have abdominal pain or signs of infectious diseases.^[3,9]^

Similar to the results of other studies,^[9,11]^ gram-positive cocci constituted more than half of the infectious organisms in effluent and blood cultures (57.7% and 63.2%, respectively) in the patients with PD peritonitis. The proportion of gram-negative bacilli was comparable in the dialysate and blood cultures (38.0% and 31.6%, respectively). *Actinobacter*, *Candida*, and *Mycobacterium* infections were discovered in a few patients, but only 1 anaerobic bacteremia was found in blood culture. Most patients with *Actinobacter*, *Candida*, and *Mycobacterium* infections have poor prognoses, and peritoneal catheter removal is imperative.^[4,11,12]^

This study has a few limitations. First, the data were collected from a single institution with limited case numbers. The results may not represent the general characteristics of patients with PD peritonitis and bacteremia. Second, 17 patients in this study did not undergo blood culture investigations in the ED, which may have altered the results of the analysis. Nevertheless, more than 90% of the study cohort had been investigated for bacteremia. The high percentage of patients undergoing blood culture investigations can eliminate the influence of this factor. Finally, all patients with PD peritonitis complicated by bacteremia had undergone both intraperitoneal and intravenous antibiotics therapies and achieved fair prognoses. The therapeutic effects of only intraperitoneal or intravenous antibiotics on these patients are still unknown.

## 5 Conclusion

The incidence of bacteremia in patients with PD peritonitis was 11.0%, and bacteremia resulted in extensive systemic derangements such as fever, tachypnea, and abnormalities in serum sodium levels. Patients with immunocompromised comorbidities carried a higher risk of developing bacteremia, which resulted in prolonged hospital stays. Combination of intraperitoneal and intravenous antibiotics therapies achieved fair prognoses in patients with PD peritonitis complicated by bacteremia.

## Author contributions

**Conceptualization:** Kuo-Tai Chen.

**Data curation:** Chang-Chih Tsai.

**Formal analysis:** Chien-Chin Hsu MD.

**Investigation:** Chang-Chih Tsai.

**Methodology:** Kuo-Tai Chen.

**Project administration:** Chang-Chih Tsai.

**Resources:** Chang-Chih Tsai.

**Software:** Kuo-Tai Chen.

**Supervision:** Chien-Chin Hsu MD.

**Validation:** Chien-Chin Hsu MD.

**Visualization:** Chien-Chin Hsu MD.

**Writing – original draft:** Chang-Chih Tsai.

**Writing – review & editing:** Kuo-Tai Chen.
